# Cost-comparison analysis of FIB-4, ELF and fibroscan in community pathways for non-alcoholic fatty liver disease

**DOI:** 10.1186/s12876-019-1039-4

**Published:** 2019-07-11

**Authors:** Ankur Srivastava, Simcha Jong, Anna Gola, Ruth Gailer, Sarah Morgan, Karen Sennett, Sudeep Tanwar, Elena Pizzo, James O’Beirne, Emmanuel Tsochatzis, Julie Parkes, William Rosenberg

**Affiliations:** 1 UCL Institute for Liver and Digestive Health, Royal Free Campus, London, NW3 2PF UK; 2 Science Based Business, Leiden University, Snellius Building, Niels Bohrweg 1, 2333 CA Leiden, Netherlands; 3 Health Economist, 6th Floor, Maple House, 149 Tottenham Court Road, London, W1T 7NF UK; 4 Department of Primary care and Population Health, Upper 3rd Floor, Royal Free Hospital, London, NW3 2PF UK; 5 Camden Clinical Commissioning Group, 75 Hampstead Rd, London, NW1 2PL UK; 6 Islington Clinical Commissioning Group Laycock St, London, N1 1TH UK; 7 Department of Applied Health Research, UCL 1-19 Torrington Place, London, WC1E7HB UK; 8 Public Health Sciences and Medical Statistics, University of Southampton, Southampton, UK

**Keywords:** Enhanced Liver fibrosis (ELF), Fibroscan, |NAFLD, Cirrhosis detection, Cost savings

## Abstract

**Background:**

The identification of patients with advanced liver fibrosis secondary to non-alcoholic fatty liver disease (NAFLD) remains challenging. Using non-invasive liver fibrosis tests (NILT) in primary care may permit earlier detection of patients with clinically significant disease for specialist review, and reduce unnecessary referral of patients with mild disease. We constructed an analytical model to assess the clinical and cost differentials of such strategies.

**Methods:**

A probabilistic decisional model simulated a cohort of 1000 NAFLD patients over 1 year from a healthcare payer perspective. Simulations compared standard care (SC) (scenario 1) to: Scenario 2: FIB-4 for all patients followed by Enhanced Liver Fibrosis (ELF) test for patients with indeterminate FIB-4 results; Scenario 3: FIB-4 followed by fibroscan for indeterminate FIB-4; Scenario 4: ELF alone; and Scenario 5: fibroscan alone. Model estimates were derived from the published literature. The primary outcome was cost per case of advanced fibrosis detected.

**Results:**

Introduction of NILT increased detection of advanced fibrosis over 1 year by 114, 118, 129 and 137% compared to SC in scenarios 2, 3, 4 and 5 respectively with reduction in unnecessary referrals by 85, 78, 71 and 42% respectively.

The cost per case of advanced fibrosis (METAVIR ≥F3) detected was £25,543, £8932, £9083, £9487 and £10,351 in scenarios 1, 2, 3, 4 and 5 respectively. Total budget spend was reduced by 25.2, 22.7, 15.1 and 4.0% in Scenarios 2, 3, 4 and 5 compared to £670 K at baseline.

**Conclusion:**

Our analyses suggest that the use of NILT in primary care can increases early detection of advanced liver fibrosis and reduce unnecessary referral of patients with mild disease and is cost efficient. Adopting a two-tier approach improves resource utilization.

## Background

The health, societal and economic burden of chronic liver disease (CLD) is substantial and represents a public health priority [1, 2]. CLD is the 5th commonest cause of death in the United Kingdom, and the only one in the top five that is increasing [3]. With rising prevalence of risk factors for liver disease including obesity and alcohol, pressure on healthcare resources is likely to intensify. Better and earlier detection of CLD in primary care is key to improving health outcomes and associated costs [4].

Non alcoholic fatty liver disease (NAFLD) is the commonest cause of deranged liver function tests (LFTs) in primary care [5]. Only a minority (5%) of these cases progresses to clinically significant liver disease [6] whilst evidence highlights fibrosis severity as the key determinant of liver related morbidity and mortality [7, 8]. The identification of patients with significant liver disease is a primary care challenge, where accurate fibrosis assessment is limited by a reliance on LFTs, which are poor discriminators of liver fibrosis [9].

In the current ‘standard care’ (SC), primary care physicians (PCP) assess the severity of a patient’s liver disease and subsequent need for specialist referral based on history, examination, blood tests including LFTs and ultrasound.

Patients referred to secondary care deemed to have significant liver disease due to NAFLD may benefit from active management (including consideration for clinical trials of emerging therapies for NAFLD and fibrosis) [2, 10]. Patients with cirrhosis will be enrolled in pathways of care that improve outcomes through targeted screening and treatment for complications of cirrhosis including portal hypertension [11] and hepatocellular carcinoma (HCC) [12]. However, current primary care approaches result in the referral of many patients that do not have significant liver fibrosis, placing a burden on secondary care services, incurring unwarranted costs and generating unnecessary inconvenience and anxiety for patients [13]. Furthermore a significant number of patients with advanced liver fibrosis are missed and not referred to secondary care. These patients have been falsely reassured and will silently progress before presenting with end-stage liver disease or liver cancer.

The use of non-invasive liver fibrosis tests (NILT) [14] may improve PCP staging of disease [4, 15] and referral practice but there is a lack of health-economic evidence about the use of NILT in fatty liver disease to inform clinicians, commissioners and policy makers about the value of such strategies. In this study, we developed a probabilistic decision analytical model to investigate the clinical and cost impact of primary care risk stratification of patients with NAFLD.

## Methods

**Fig. 1:**
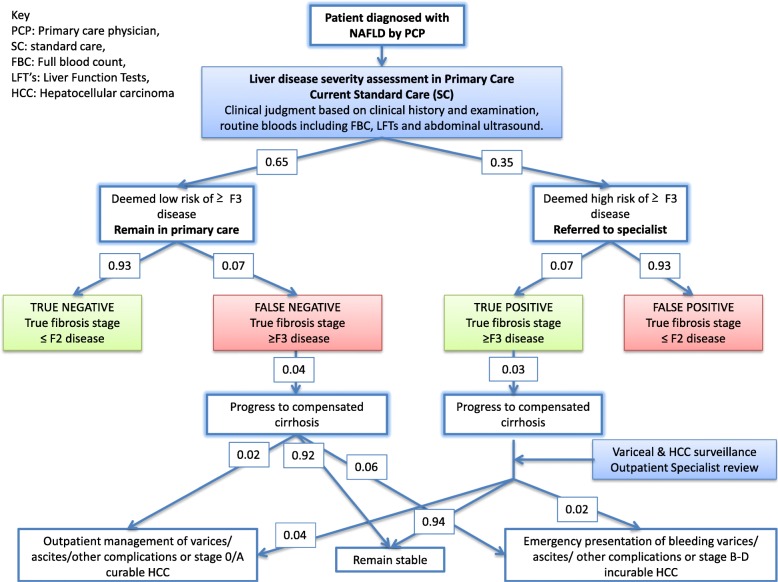
Schematic simulating current ‘standard of care’ patient journey. Simplified simulated journey of a patient with NAFLD through the healthcare system after primary care assessment using standard of care over a 1-year timeframe (see Table 1 and Table 2 for references). The diagnostic performance of the primary care assessment has four outcomes - *1) ‘True positive’; Patients deemed to be at high risk for advanced fibrosis subsequently confirmed as having ≥ F3 fibrosis after specialist assessment.* Patients will be actively managed in secondary care (including consideration for clinical trials). Patients with cirrhosis will be enrolled in pathways of care that improve outcomes through targeted screening and treatment for portal hypertension and hepatocellular carcinoma (HCC). 2) *‘True negative’; Patients deemed to be at low risk for advanced fibrosis found to have ≤ F2 disease.* These patients are unlikely to suffer morbidity from their liver disease. Management in primary care should be focussed on managing reversible metabolic disorders. 3) *‘False positive’; Patients deemed to be at high risk for advanced fibrosis in primary care but found to have ≤ F2 fibrosis.* The pathway can be considered to have failed this group of patients, whom can be managed effectively in primary care with weight loss and exercise. 4) *‘False negative’; Patients deemed to be at low risk for advanced fibrosis who have ≥ F3 fibrosis.* This cohort of patients have been falsely reassured and represent a failure of the pathway as they remain in primary care unless they present with complications of CLD if their disease progresses, at which point interventions are increasingly limited

A liver working group, comprising primary care physicians and secondary care liver specialists, commissioners, public health practitioners and patient representatives was formed in the London Boroughs of Camden and Islington to develop new pathways of care for patients with NAFLD. Part of the strategy was to establish current practice (standard care (SC)) (Fig. 1) and to establish pragmatic guidance on how to improve the identification of NAFLD cases with advanced liver disease for referral to secondary care. In the first instance, PCP favoured the selection of patients with deranged liver function tests (LFT) even though it was agreed that this would miss a minority of cases with liver fibrosis who have normal LFTs.

### Probabilistic decision model

**Fig. 2:**
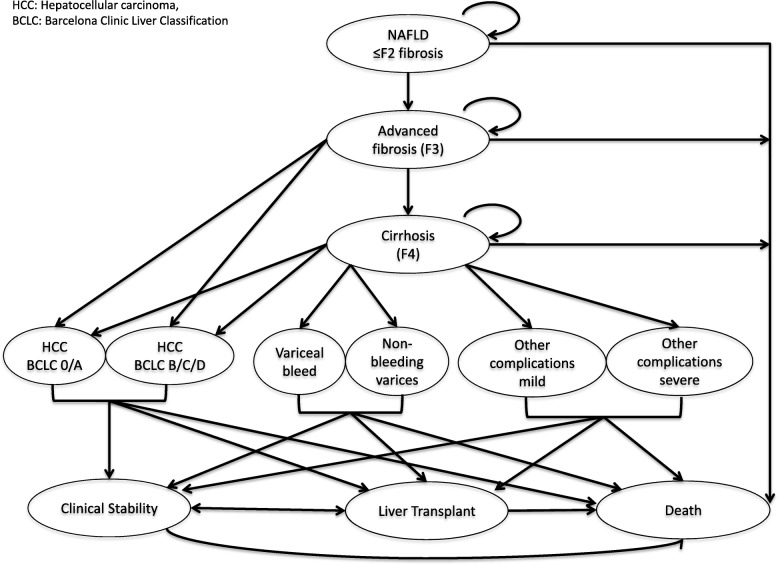
Decision tree presenting overview of transition of a patient with NAFLD through the model. In the model, a patient with NAFLD and ≤ F2 fibrosis could remain well, progress to F3 fibrosis or die. A patient with F3 fibrosis could remain well, progress to compensated cirrhosis, develop HCC or die. Patients with compensated cirrhosis could remain stable, develop a complication of cirrhosis, undergo liver transplantation or die. The model differentiated early stage complications (non-bleeding varices detected by surveillance endoscopy, Barcelona Clinic Liver Cancer (BCLC) stage 0/A HCC and mild/moderate ‘other’ complication including ascites, jaundice and hepatic encephalopathy managed as outpatient), from late stage complications (bleeding varices, BCLC stage B-D HCC and severe ‘other’ CLD complications necessitating inpatient admission)

A probabilistic decision analytical simulation model was created using Microsoft Excel Software (version16.23, 2019). The model piloted competing primary care risk stratification diagnostic strategies for 1000 patients with a confirmed diagnosis of NAFLD **(**Fig. 2**).** The average patient was 50 years old with elevated transaminases. The cycle length was 1 year.

### Competing Strategies in the Model and Analyses

We modelled the standard care in the UK National Health Service (NHS) (scenario 1). The use of FIB-4 and ELF in a two-tier stratification approach (scenario 2) was modelled to replicate a local pilot pathway - the Camden and Islington NAFLD pathway [13]. Following an independent evaluation of NILT public health consultants favoured the use of FIB-4 over the NAFLD Fibrosis Score, in part due to a lack of standardization in the diagnosis of diabetes. Fibroscan is increasingly established in secondary care practice, and was incorporated to assess its performance in place of ELF in a two-tier strategy (Scenario 3). One-tier approaches were also considered in which SC was supported by ELF (scenario 4), or fibroscan alone (Scenario 5).

In all scenarios SC delivered by PCPs included history, physical assessment followed by investigation of liver function, tests for viral, immune and metabolic causes of liver disease and an ultrasound scan in order to make an assessment of the risk of advanced liver fibrosis. This was classified as a binary outcome with the case deemed to be at ‘high risk’ of advanced liver disease necessitating referral to a specialist, or at ‘low risk’ and thus appropriate for management in primary care. This decision process required 3 PCP consultations, 3 routine bloods tests and 1 ultrasound scan. In scenarios 2 and 3, SC was supported by the calculation of a FIB-4 score in all patients to improve the identification of patients at risk of advanced fibrosis (METAVIR ≥ F3). Low risk patients (FIB-4 < 1.30) were managed in primary care whilst high-risk patients (FIB-4 > 3.25) were referred to a specialist in secondary care. Patients with indeterminate scores (FIB-4 1.30–3.25) required an ELF test (Scenario 2) or a community fibroscan (Scenario 3). Published cut-offs were used to identify cases at increased risk of advanced fibrosis (≥10.3 for ELF and ≥ 7.9 kPa for Fibroscan). A fibroscan failure rate of 5% was assumed [47]. For patients managed in Scenario 4, SC was followed by ELF test for all patients and in Scenario 5 SC was followed by fibroscan for all patients.

Patients identified as being at high-risk of advanced fibrosis were referred to a secondary care specialist. Evaluation included further blood tests, fibroscan, imaging including US scan (50% of cases, informed by local audit), CT scan (5% of cases, informed by local audit), MRI Liver (5%, informed by local audit) and liver biopsy (15% of cases, informed by local audit). Patients deemed to not have advanced fibrosis (false positive) would be discharged to primary care, whilst those confirmed with advanced fibrosis would enter recognised surveillance pathways.

The time horizon for the base-case was 1 year to assess short-term benefits, likely to relate to resource utilisation. A 5- year timeframe was applied to assess the longer-term implications.

### Clinical data inputs

A comprehensive literature search informed model parameters. The data were critically assessed to ensure suitability for this study and were supplemented by expert opinion when required.

**Fig. 3:**
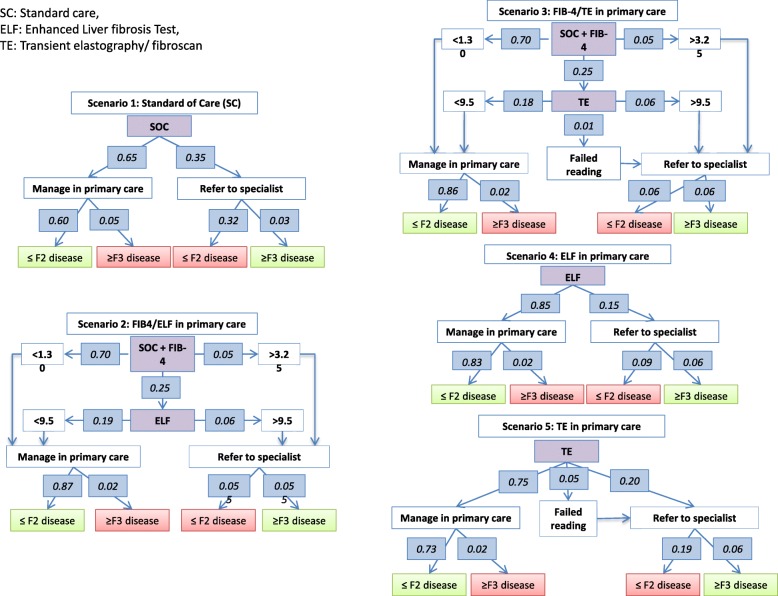
Flow diagram depicting patient flow in each simulated scenario**.** Published test performances allowed prediction of true and false positive and negative rates for detection of advanced fibrosis (≥F3) in each scenario

The model assumed an intention-to-diagnose strategy. All patients would be managed according to the pathways. SC performance is poorly documented, but estimates were extrapolated from available studies [13, 16, 17]. Advanced fibrosis (≥F3 fibrosis) prevalence was set at 7.5% [5] and published sensitivities and specificities of FIB-4 [18, 19], ELF [19, 20] and fibroscan [19, 21] were used to predict stratification rates for low- and high- risk of advanced fibrosis (Table 1 and Fig. 3). Estimates of NAFLD disease progression were used to inform pathway performance (Table 2).

**Table 1:** Test performance and Disease prevalence estimates

Test characteristics	Sensitivity	Specificity	Reference
Standard of care	0.35	0.65	Expert Opinion [16, 17]
FIB-4 (cut off 1.30)	0.84	0.74	[18, 19]
FIB-4 (cut off 3.25)	0.38	0.97	[18, 19]
ELF	0.80	0.90	[19, 20]
Fibroscan	0.82	0.84	[19, 21]
Population and disease characteristics	Transition probability		
Prevalence of advanced fibrosis in the general population	0.075		[5]

Published test characteristics of non-invasive liver fibrosis tests to detect advanced fibrosis (METAVIR ≥F3) in patients with non-alcoholic fatty liver disease

**Table 2:** Transitional probability estimates used to populate the probabilistic analytical model for the base case (annual progression rates)

Parameter/ Health state	Transition probability		References
Population and disease characteristics			
Prevalence of advanced fibrosis in the general population	0.075		[5]
Reduction in fibrosis progression after GP management	0.01		[22], Author assumption
Reduction in fibrosis progression after specialist review	0.025		[22]
Mild/ No fibrosis (F0, F1, F2)			
Remain healthy	0.99		[23–26]
Develop F3 disease	0.001		[23, 24]
Mortality (all cause)	0.005		[25, 27, 28]
Discharge from specialist services	0.7		Unpublished audit
Advanced fibrosis (F3)			
Remain healthy	0.95		[25, 29, 30]
Develop F4 disease/ cirrhosis	0.04		[25, 29, 30]
Develop HCC (without cirrhosis)	0.004		[29]
Mortality (all cause)	0.005		[29]
Compensated cirrhosis (F4)			
Remain compensated	0.93		Calculated from other variables
Develop varices	0.03		[31]
Develop HCC	0.003		[32, 34–37]
Develop other complications (inc. jaundice, ascites, HE)	0.02		[29, 31, 32]
Mortality (all cause)	0.02		[31, 38, 39], expert opinion
BCLC Stage 0 and A HCC			
Cure (liver transplant)	0.36		[40]
Cure (non transplant)	0.39		[40]
Mortality (all cause)	0.25		[40]
BCLC stage B – D HCC			
Clinical stability (post TACE, RFA etc)	0.24		[41]
Mortality (all cause)	0.76		[35]
Varices detection in surveillance programme			
Clinical stability	0.92		[42]
Liver transplant	0.01		Expert opinion
Mortality (all cause)	0.07		[33]
Detection of varices after emergency presentation			
Clinical stability	0.73		[43]
Liver transplant	0.02		[42]
Mortality (all cause)	0.25		[44]
Mild/ Moderate ‘other’ complication			
Clinical stability	0.74		[17]
Liver transplant	0.10		[42]
Mortality (all cause)	0.16		[17]
Severe ‘other’ complication			
Clinical stability	0.45		[17]
Liver transplant	0.10		[42]
Mortality (all cause)	0.45		[17]
Severity of CLD Complication	No screening	Screening	
Probability of BCLC stage 0 + A HCC	0.299	0.709	[45]
Probability of BCLC stage B - D HCC	0.701	0.291	[45]
Detecting varices in surveillance programme	0.0	0.60	[42]
Detecting varices after emergency presentation	100.0	0.40	[42]
Mild/ moderate CLD ‘other’ complication	0.527	0.622	[17, 46]
Severe CLD ‘other’ complication	0.473	0.378	[17, 46]

### Cost data inputs

A healthcare payer perspective was adopted. Costs were derived from published resources and local costing tariffs (February 2015) (Table 3) for the UK. A 3.5% discount rate was applied. Direct healthcare costs included PCP consultations, blood tests and ultrasound scans. The cost of FIB-4 was considered to be £0, as ALT, AST and platelet tests were incorporated as ‘routine blood tests’. ELF was priced at £42, the quoted rate charged to the Camden and Islington CCG in their pathway, and fibroscan was priced at £43 [19]. In secondary care, the costs incurred related to specialist consultations, investigations including liver biopsy and expenditure related to HCC surveillance (6 monthly AFP and ultrasound) and variceal surveillance (2–3 yearly endoscopy). Finally, costs for the management of complications of CLD, including inpatient and outpatient costs, pharmacological treatment and surgical procedures including liver resection and transplant were obtained from the Royal Free London NHS Foundation Trust finance department.

**Table 3:** Health care costs for patients with NAFLD/ NASH (£, 2014–2015)

Resource Use	Unit Cost	Reference
Primary Care		
GP consultation (per patient contact lasting 11.7 min)	£45.00	[48]
Secondary care		
Hepatology Consultant appointment (new)	£148.34	Royal Free, February 2015
Hepatology Consultant follow up appt	£98.63	Royal Free, February 2015
Dietician review	£57.00	Royal Free, February 2015
Investigations		
Routine blood tests (inc. FBC, LFT’s, INR)	£68.06	Royal Free, February 2015
Liver aetiology panel	£147.98	Royal Free, February 2015
FIB-4 (AST/ALT/ platelets included in ‘routine blood tests’)	£0.00	Royal Free, February 2015
ELF	£42.00	North Middlesex Hospital, February 2015,
Ultrasound Liver	£63.67	Royal Free, February 2015
CT Abdomen/ Liver	£80.78	Royal Free, February 2015
MRI Abdomen/ Liver	£101.00	Royal Free, February 2015
Fibroscan	£43.00	Royal Free, February 2015
Liver biopsy	£642.75	Royal Free, February 2015
Endoscopy	£264.00	Royal Free, February 2015
Surgical procedures		
Liver resection	£7000	[49]
Liver transplant (1st year)	£70,000	[19, 49, 50], Royal Free, February 2015

The primary outcome measure was cost per case of advanced fibrosis detected - a surrogate for cost utility.

Secondary outcomes included unnecessary referral rates of patients with non-advanced disease, the severity of CLD complications, liver transplantation and mortality rates.

## Results

### Clinical outcome

The base case analysis for 1000 patients with NAFLD over a 1-year timeframe demonstrated 650 patients (65%) were identified as being at low risk of advanced fibrosis and remained in primary care (scenario 1, SC). Of this group, 49 patients (8%) had advanced fibrosis but remained in primary care inappropriately (false negative rate). The remaining 350 patients (35%) were referred to a specialist. After specialist investigation, 93% (324 patients) were determined to be at low risk (false positive rate) and discharged whilst 26 patients (7%) were confirmed to have advanced fibrosis (true positive) and progressed on specialist pathways.

**Fig. 4:**
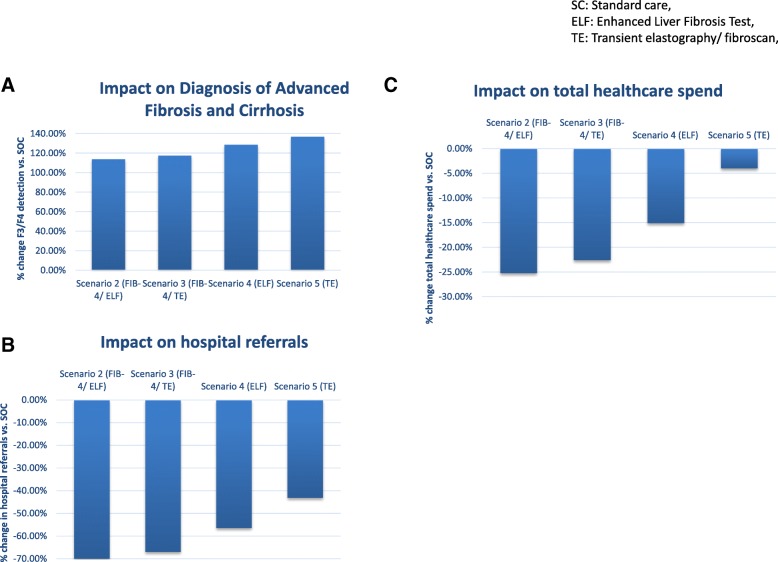
Clinical impact of risk stratification in primary care**.** Graphs illustrating the impact of introducing NILT into primary care on increasing identification of advanced fibrosis/ cirrhosis (**a**), reducing hospital referrals (**b**) and reducing overall healthcare spend (**c**), for 1000 NAFLD patients over 1 year

The impact of introducing non-invasive tests into primary care using FIB-4 and ELF (Scenario 2), FIB-4 and TE (Scenario 3), ELF alone (scenario 4) or TE alone (Scenario 5) was assessed (Fig. 4 and Table 4). Over the 1 year time-horizon, compared to SC these strategies reduced the relative referral rate from primary care to hospital by 70, 67, 56 and 43% for scenarios 2, 3, 4 and 5 respectively; corresponding to 245, 223, 198 and 150 fewer referrals over 1 year per 1000 patients. This reduced the need for investigation performed in secondary care. The number of patients requiring imaging in secondary care reduced by 147, 134, 118 and 60 in scenarios 2, 3, 4, and 5 respectively, whilst 25, 22, 20 and 10 fewer patients required endoscopy after 1 year per 1000 patients referred. The requirement for liver biopsy was reduced by 37, 33, 30 and 15 patients in scenarios 2, 3, 4 and 5 respectively. This translated into cost savings in secondary care investigation in the first year per 1000 patients referred of £165,530.04, £150,184.67, £133,505.60 and £68,256.85 for scenarios 2, 3, 4 and 5 respectively.

**Table 4:** Base case analysis of introducing FIB-4, ELF and fibroscan into primary care risk stratification pathways compared to standard of care (scenario 1) after 1 year for 1000 patients with NAFLD

	Scenario 2 - FIB-4/ELF	Scenario 3 - FIB-4/ TE	Scenario 4 - SOC + ELF	Scenario 5 - SOC + TE
Pathway performance: patients referred to specialist (secondary care)				
Incremental number of referrals (stratified as ≥F3 fibrosis) (% increase vs SOC)	−245 (− 70%)	− 222 (− 67%)	−198 (− 56%)	− 101 (25%)
Incremental number of ≥F3 disease referred	30 (53%)	31 (45%)	34 (39%)	36 (25%)
Incremental number of ≤F2 disease referred	−275 (−85%)	−253 (−78%)	− 231 (−71%)	−137 (−42%)
Incremental number of cirrhotics referred	1.16 (113%)	1.20 (116%)	1.31 (128%)	1.40 (136%)
Pathway performance: patients remain under primary care management				
Incremental number of patient stratified as ≤F2 fibrosis (Primary care management)	245 (38%)	222 (34%)	198 (30%)	101 (15%)
Incremental number of patients correctly identified as ≤F2	274 (46%)	253 (42%)	231 (38%)	137 (23%)
Incremental number of patients incorrectly identified as ≤F2	−30 (−61%)	−31 (−63%)	−34 (− 69%)	−36 (−74%)
Overall performance of pathways				
Sensitivity	0.75	0.76	0.80	0.83
Specificity	0.95	0.92	0.90	0.80
Positive Predictive Value	0.53	0.45	0.39	0.25
Negative Predictive Value	0.98	0.98	0.98	0.98
Positive Likelihood Ratio	14.11	9.96	8.00	4.10
Negative Likelihood ratio	0.27	0.26	0.22	0.21
Impact on end stage liver disease				
BCLC Stage 0/A curable HCC (% of all HCC)	0.06 (36%)	0.06 (38%)	0.07 (41%)	0.08 (44%)
BCLC Stage B-D incurable HCC (% of all HCC)	−0.06 (−29%)	−0.07 (−30%)	−0.07 (−33%)	−0.08 (−35%)
Varices detected via surveillance programme (% of all new varices)	0.02 (113%)	0.02 (117%)	0.02 (128%)	0.03 (136%)
Emergency presentation of varices (% of all new varices)	−0.02 (−30%)	− 0.02 (−31%)	−0.02 (−34%)	−0.03 (−36%)
Mild/Moderate ‘other’ complications	< 0.01 (6%)	< 0.01 (6%)	< 0.01 (7%)	< 0.01 (7%)
Severe ‘other’ complication	< 0.01 (−9%)	< 0.01 (−9%)	< 0.01 (−10%)	< 0.01 (−10%)
Number of liver transplants (of all cirrhotics known to specialist)	0.02 (32%)	0.02 (33%)	0.03 (36%)	0.03 (39%)
Outcomes				
Mortality / 1000 NAFLD patients	−0.03 (−0.34%)	− 0.03 (− 0.35%)	−0.04 (− 0.39%)	−0.04 (− 0.41%)

Tabulated analysis of the impact of non-invasive liver fibrosis tests for the management of patients with NAFLD (scenarios 2–5) compared to the standard of care (scenario 1) in the primary care setting

These approaches resulted in reductions in referral of patients with non-advanced liver fibrosis (deemed “unnecessary” referrals) by 85, 78, 71 and 42% (absolute reduction) in scenarios 2, 3, 4, and 5 respectively compared to scenario 1; corresponding to 275, 253, 231 and 137 reduction in inappropriate referrals from 324 patients in scenario 1 over the 1-year time horizon.

TE alone was the most clinically effective strategy in the detection of ≥F3 fibrosis. Compared to SC, introducing NILT increased the identification of patients with advanced fibrosis (true positive rate) by 30, 31, 34 and 36 patients in scenarios 2, 3, 4 and 5 respectively equating to an 114, 118, 129 and 137% improvement..

Considering cirrhosis specifically, over the 1-year timeframe, employing each of the strategies improved detection by 1 patient per 1000 population compared to the SC,. Specifically, an extra 1.2 (113%), 1.2 (116%), 1.3 (128%) and 1.4 (136%) cirrhotic patients per 1000 population were detected in scenarios 2, 3, 4 and 5 respectively over 1 year compared to SC.

The model tested the impact of earlier detection of advanced fibrosis on complications of CLD (Table 4). Patients known to have cirrhosis routinely undergo regular surveillance. The model demonstrated that the number of patients presenting with curable HCC (Stage O/A) increased from a base case of 0.17 patients per 1000 population following SC, by 0.06 patients per 1000 population in scenarios 2 and 3 and by 0.07 and 0.08 patients per 1000 population in scenarios 4 and 5 respectively. Conversely, over 1 year, incurable HCC (stage B-D), rates decreased from a base case of 0.22 patients per 1000 population following SC the same amounts equivalent to reductions of 29, 30, 33 and 35% for scenarios 2–5 respectively. Patients presenting with variceal haemorrhage reduced by 0.02 patients per 1000 population in scenarios 2, 3 and 4, and by 0.03 patients per 1000 population in scenario 5, compared to SOC (0.07 patients per 1000 population). The model predicted that variceal detection through surveillance prior to haemorrhage increased from 0.02 patients per 1000 with SC by 0.02 patients per 1000 in scenarios 2, 3, 4 and 0.03 patients per 1000 in scenario 5, permitting the instigation of primary prophylaxis of variceal haemorrhage that is associated with reduced mortality [44]. Finally, the model demonstrated that management of cirrhosis in secondary care achieved a reduction in episodes of hospitalization due to other complications of CLD including jaundice, ascites and hepatic encephalopathy from 0.03 patients per 1000 in SC by 0.01 patients per 1000 in all scenarios.

Improvements in the detection and management of cirrhosis would permit increased rates of liver transplantation by 0.02 patients per 1000 in scenarios 2 and 3 and 0.03 patients per 1000 in scenarios 4 and 5, compared to SC (0.07 patients per 1000) over 1 year. Predicted all-cause mortality reduced by 0.03 patients per 1000 in scenarios 2 and 3 and 0.04 patients per 1000 in scenarios 4 and 5 over 1 year compared to SC (9.87 patients per 1000).

### Cost outcome

**Fig. 5:**
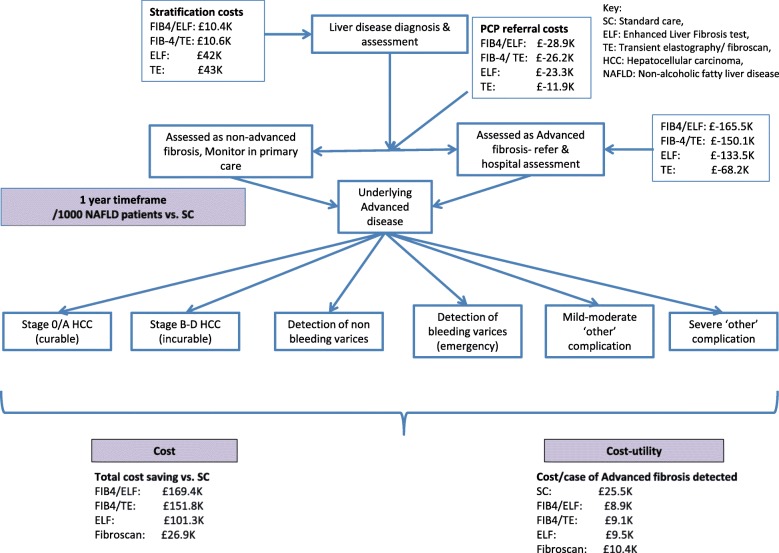
Cost impact of risk stratification in primary care**.** Incremental cost expenditure and savings compared to SOC at different stages of the management pathway over a 1-year time horizon for 1000 NAFLD patients

The healthcare costs of the competing strategies are summarised in Fig. 5 using a 1-year horizon. For 1000 patients with NAFLD undergoing PCP assessment for liver disease, the costs directly associated with NILT were £10,385 in Scenario 2, £10,632 in scenario 3, £42,000 in scenario 4 and £43,000 in scenario 5 (incorporating additional PCP appointments and blood tests).

Compared to SC (scenario 1) which cost £670,504 over 1 year, the incremental reductions in healthcare spending achieved through use of NILT in each scenario were £169 K, £152 K, 101 K and 27 K per 1000 patients in 1 year in scenarios 2, 3, 4 and 5 respectively equating to reductions of 25, 23, 15 and 4%. Using cost-per-case of advanced fibrosis as a surrogate for cost utility, all scenarios were favourable to SC (£25,543.02), with the model predicting cost-per-case of advanced fibrosis at £8.932.19, £9083.78, £9487.26 and £10,351.67 in scenarios 2, 3, 4 and 5 respectively over the 1 year timeframe**.**

From a commissioning perspective, a significant contributor to the immediate cost saving was the reduction in secondary care referrals. Compared to scenario 1 (£41,300 per 1000 patients over 1 year), cost-savings attributable to reduced specialist referral were £28,895, £26,216, £23,305 and £11,915 in scenarios 2, 3, 4 and 5 respectively, equating to 70, 63, 56 and 29% reductions.

A budget impact analysis (Table 5) assessed the impact of introducing the interventions nationally in the UK NHS system. Assuming the prevalence of NAFLD to be 20% in the UK population of 60 million people and a 5-year cycle of disease assessment, nationally 2.4 million people would potentially be risk stratified annually. The incremental cost to the budget holder of introducing NILT for fibrosis in NAFLD in primary care would be £24.9 M, £25.5 M, £100.8 M and £103.2 M in scenario’s 2, 3, 4 and 5 respectively. However, this would deliver savings equating to 23, 21, 14 and 4% reductions in total healthcare expenditure for each of the scenarios respectively.

**Table 5:** Budget impact analysis of introducing FIB-4, ELF and fibroscan into primary care risk stratification pathways compared to standard care after 1 year for a population of 60 million patients

	Scenario 2 - FIB-4/ELF	Scenario 3 - FIB-4/ TE	Scenario 4 - SC + ELF	Scenario 5 - SC + TE
Pathway performance:				
Incremental number of referrals (stratified as ≥F3 fibrosis) (% increase vs SOC)	− 587,700 (−70%)	− 533,217 (−67%)	− 474,000 (−56%)	− 242,340 (−25%)
Incremental number of ≥F3 disease referred	71,640 (53%)	74,041 (45%)	81,000 (39%)	86,220 (25%)
Incremental number of ≤F2 disease referred	− 659,340 (−85%)	− 607,258 (−78%)	− 555,021 (−71%)	− 328,560 (−42%)
Incremental number of cirrhotics referred	2786 (113%)	2880 (116%)	3153 (128%)	3359 (136%)
Incremental number of patients incorrectly identified as ≤F2	−71,640 (−61%)	−74,041 (− 63%)	−81,000 (− 69%)	−86,220 (− 74%)
IMPACT ON END STAGE LIVER DISEASE				
BCLC Stage 0/A curable HCC (% of all HCC)	121 (36%)	125 (38%)	137 (41%)	146 (44%)
BCLC Stage B-D incurable HCC (% of all HCC)	−121 (− 29%)	− 125 (− 30%)	−137 (− 33%)	− 146 (− 35%)
Varices detected via surveillance programme (% of all new varices)	50 (113%)	51 (117%)	57 (128%)	60 (136%)
Emergency presentation of varices (% of all new varices)	−50 (− 30%)	−51 (− 31%)	−57 (− 34%)	−60 (− 36%)
Mild/Moderate ‘other’ complications	5 (6%)	5 (6%)	6 (7%)	< 6 (7%)
Severe ‘other’ complication requiring hospital admission	−5 (− 9%)	− 5 (− 9%)	− 6 (− 10%)	−6 (− 10%)
Outcomes				
Mortality / 1000 NAFLD patients	−67 (− 0.34%)	−69 (− 0.35%)	−76 (− 0.39%)	−81 (− 0.41%)
Budget				
Cost of tests	£24.9 M	£25.5	£100.8	£103.2 M
Total expenditure	-£406 M (−23%)	-£364 M (−21%)	-£243 M (−14%)	-£65 M (− 4%)

Tabulated analysis of the impact of non-invasive liver fibrosis tests for the management of patients with NAFLD (scenarios 2–5) compared to the standard of care (scenario 1) in the primary care setting for a population of 60 million patients with 20% NAFLD prevelance risk stratified on a 5 year cycle

Table 6 summarises the outcomes of introducing non-invasive liver fibrosis tests in primary care for the base case.

**Table 6:** Summary of outcomes resulting from introducing non-invasive liver fibrosis tests in primary care (per 1000 NAFLD patients over 1 years)

	Scenario 1 Standard Care	Scenario 2 FIB 4 +/− ELF	Scenario 3 FIB 4 +/− TE	Scenario 4 ELF	Scenario 5 TE
Total Referrals avoided (vs. SOC)	–	245	234	198	150
Cases F3/F4 detected	26.3	56.1	57.1	60.0	62.2
Cases Cirrhosis detected	5.3	11.0	11.2	11.8	12.3
Cases Cirrhosis missed	11.3	5.5	5.3	4.7	4.2
Cost saving (vs. SOC)	–	- £169,408	- £151,816	- £101,268	- £26,889
Cost per ≥ F3 detected	£25,543	£8932	£9083	£9487	£10,351

### Projected outcomes over a 5 year timeframe

Analyses were performed using a five-year horizon to assess the longer-term outcomes of the pathway (Table 7).

**Table 7:** Projected clinical outcomes and costs of the scenarios projected over 1 year and 5 years

	Scenario 1- SC		Scenario 2 - FIB-4/ELF		Scenario 3 - FIB-4/ TE		Scenario 4 - SC + ELF		Scenario 5 - SC + TE	
	1 year	5 years	1 year	5 years	1 year	5 years	1 Year	5 years	1 year	5 years
Total number of cirrhotics entered into specialist services (out of all cirrhotics)	1.03 (34%)	5.28 (32%)	2.19 (74%)	10.97 (66%)	2.23 (75%)	11.17 (68%)	2.34 (79%)	11.75 (71%)	2.43 (82%)	12.23 (74%)
Total number of cirrhotics not known to specialist services (out of all cirrhotics)	1.96 (66%)	11.278 (68%)	0.78 (26%)	5.53 (34%)	0.74 (25%)	5.41 (32%)	0.63 (21%)	4.73 (29%)	0.54 (18%)	4.24 (26%)
Early stage complication (stage 0/A HCC, non-bleeding varices, mild ascites etc) (% of all complications) Cost	0.22 (42%) £3.0 K	3.52 (39%) £31.1 K	0.31 (57%) £4.1 K	4.62 (52%) £41.2 K	0.31 (58%) £4.1 K	4.66 (52%) £41.5 K	0.32 (60%) £4.3 K	4.77 (54%) £42.6 K	0.33 (61%) £4.3 K	4.87 (55%) £43.4 K
Late stage complication (stage B-D HCC, bleeding varices, severe ascites etc) (% of all complications) Cost	0.32 (58%) £12.9 K	5.44 (61%) £141 K	0.23 (43%) £9.2 K	4.3 (48%) £108 K	0.23 (42%) 9.1 K	4.26 (48%) £107 K	0.22 (40%) £8.8 K	4.14 (46%) £103 K	0.21 (39%) £8.4 K	4.05 (45%) £101 K
Liver transplant Cost	0.07 £5.9 K	1.05 £89.5 K	0.10 £7.9 K	1.16 £98.9 K	0.10 £8.0 K	1.16 £99.3 K	0.10 £8.2 K	1.17100.2 K	0.10 £8.3 K	1.18 £101 K
Mortality (%)	9.87 0.99%	28.56 2.86%	9.84 0.98%	28.18 2.82%	9.83 0.98%	28.17 2.82%	9.83 0.98%	28.13 2.81%	9.83 0.98%	28.10 2.81%
Total cost/1000 NAFLD patients	638 K	1.1 M	502 K	946 K	522 K	971 K	570 K	1.0 M	647 K	1.1 M
Cost/advanced fibrotic detected	25.7 K	49.9 K	9.0 K	19.4 K	9.1 K	19.4 K	9.5 K	19.5 K	10.4 K	20.5 K

Five years after the introduction of NILT the model demonstrated clinical benefit, with increases in the detection of cirrhosis by 107, 111, 123 and 132% in scenarios 2, 3, 4 and 5 respectively equating to an extra 5.69, 5.90, 6.48 and 6.95 cases per 1000 patients tested per year.

Using a discount rate of 3.5%, compared to SC over 5 years incremental savings of £168,449.80, £142,752.51, £86.604.60 and £20,769.62 were made in scenarios 2, 3, 4, and 5 respectively.

### One way sensitivity analyses

We performed a one-way sensitivity analysis on the base-case scenario using a time-frame of 1 year.

A pathway uptake sensitivity analysis tested assumptions about the proportion of patients entering the pathway (0–100%) and confirmed a linear benefit proportional to pathway uptake and reinforced that any utilisation of the pathway (i.e. > 0%) would deliver benefit in all scenarios over the 1-year timeframe.

A clinical effectiveness sensitivity analysis was performed by varying the specificity of SC for the detection of advanced fibrosis. In the base-case model, a value of 0.65 was assumed (65% specificity for the detection of advanced fibrosis). To counter the influence of this assumption, around which sparse data are published, it was varied from 0.00 to 1.00, demonstrating a significant influence on cost-effectiveness. However, the cost-benefit was only negated when the specificity of SC for the detection of advanced fibrosis exceeded 0.88, 0.86, 0.80 and 0.68 in scenarios 2, 3, 4 and 5 respectively.

## Discussion

Our cost consequence analyses indicate that the use of NILT to stratify patients with NAFLD in primary care is clinically effective and cost saving. Utilizing fibroscan alone was most effective in detecting patients with advanced fibrosis, whilst employing FIB-4 and ELF delivered the greatest cost saving.

All of the scenarios using NILT in primary care permitted the earlier identification of advanced fibrosis/cirrhosis, creating opportunities to modify fibrosis progression [22, 51] and to start surveillance and treatment of varices and HCC. Modelling indicated that significant benefits could accrue from the detection of early stage curable HCC (stage 0/ A) and non-bleeding varices that can be treated with beta-blockers and band ligation that can avert emergency presentations with bleeding varices. A modest reduction in hospital admissions for other complications of CLD including jaundice, ascites and hepatic encephalopathy was demonstrated. The relatively limited impact of current care pathways on mortality, largely attributable to missed or late diagnosis of advanced fibrosis, highlights the need for new approaches to diagnosis and treatment to prevent NAFLD progression.

Of interest to commissioners, the implementation of NILT in primary care offers the potential to reduce the total number of referrals, and in particular the unnecessary referral of patients who have minimal fibrosis. Over a 1-year horizon, there was a reduction in total referrals of 70, 63, 56 and 29% in scenarios 2, 3, 4 and 5 respectively, with an 85, 78, 71 and 42% reduction in referrals of patients with non-advanced disease. Real-world data from a secondary care hepatology service found that 66% of referred patients had a baseline FIB-4 score < 1.30 indicating that these patients had a low risk of advanced fibrosis and could have avoided referral [13]. This group of patients represents an inefficient use of resources, adding pressure to overstretched outpatient specialist services and PCP healthcare budgets [52]. Introduction of the use of NILT in primary care would deliver immediate reductions in expenditure through avoidance of unnecessary referrals, unlike the cost-benefits associated with improved outcomes attributable to earlier diagnosis that accrue much later.

All scenarios were cost saving. The cost of detecting a case of advanced fibrosis using SC was £49,917.83 in scenario. This compared to £19,360.75 in scenario 2, £19,448.49 in scenario 3, £19,487.75 in scenario 4 and £20,451.35 in scenario 5. Healthcare budgets were reduced by 17, 15, 11 and 3% in scenarios 2, 3, 4 and 5 respectively, attributable to the reduction in costs associated with end stage liver disease and improved resource utilisation. The budget decrease in scenario 5 was modest as it was assumed all patients who failed fibroscan (5% of cohort) were referred to specialists.

In this analysis, we explored the impact of the use of FIB-4 and ELF to replicate a primary care pathway introduced in north London. This combination of tests was optimal for the stratification of patients in a hospital based population [53] and has subsequently been shown to be clinically effective [13] when applied in primary care where the proportion of cases of advanced fibrosis amongst cases of NAFLD is smaller.

There are limitations to the model. The model was populated with the best available published evidence. A lack of high quality data for some variables was remedied with expert opinion. The measures of performance for FIB-4, ELF and fibroscan were drawn from validations in hospital cohorts, but these estimates may be inappropriately high for primary care populations in which the prevalence of fibrosis is likely to be lower (spectrum bias) [54, 55]. The model assumes that use of ELF and fibroscan as second-tier tests has the same performance characteristics as a first-tier test. This may under-estimate the performance of the pathway. Additionally, the model was limited to FIB-4, ELF and fibroscan. Local PCP focus groups supported by consultants in Public Health, formed to aide Camden and Islington pathway development, outlined the practical need for simplicity, giving FIB-4 an advantage over the NAFLD-fibrosis score, which the PCP considered to be more challenging to obtain such as BMI, or associated with uncertainty about case definition such as diabetes. The model examines fibrosis, but not NASH, and so may underestimate disease progression. Other sources of error include analytical performance. We assumed a 5% failure rate for fibroscan but higher rates have been reported [47], whilst serum tests can be influenced by comorbidities. For the purposes of the model, liver biopsy was considered the reference test for liver fibrosis. Given the inherent inaccuracies associated with liver biopsy [56], this may have over-estimated the performance of liver biopsy. The cost estimate of liver biopsy incorporated procedural elements but not those associated with complications of the procedure. This approach may underestimate the true cost associated with liver biopsy. The clinical- and cost- efficiency of all scenarios are highly favourable, but may not reflect real-life outcomes. Not all patients with NAFLD consult their PCP, and pathway uptake by health professionals is variable. The base case is a 50-year-old man with abnormal transaminases, reflecting a screening strategy identified by the Camden and Islington steering committee as practical in real-life primary care practice. However this approach will miss cases of NAFLD with advanced fibrosis but normal LFTs. A screen-all strategy is likely to be clinically optimal, as patients with NAFLD and normal transaminases are at risk of significant fibrosis. The costing in the model is comprehensive, assuming full adherence to guidelines and protocols and thereby potentially overestimating the cost of care. We employed a probabilistic decision model as our main economic focus was on the payer perspective rather than a population health perspective, where alternative cost-effectiveness approaches using quality of life data and Markov simulations would be desirable. The lack of beta or triangular distributions and true probability sensitivity analysis limits the model. The model lacks cost/ QALY data and relies on descriptive measures including cost per case of advanced fibrosis detected. These outcome measures have no standard comparator limiting the current model. These shortcomings will be addressed in future work.

NAFLD should be considered as the hepatic manifestation of a multisystem disorder associated with widespread morbidity including cardiovascular disease, diabetes, hyperlipidaemia and cancer. While a fully comprehensive health economic model would need to take these morbidities into consideration we chose to focus on liver disease. Accommodating all NAFLD associated morbidities, their evaluation and management was beyond the scope of this study.

This study has several strengths. A comprehensive literature review was undertaken to identify estimates for clinical parameters, transition rates and costs. The study adds to the current body of evidence and our conclusions are similar to other health economic analyses [57]. A Health Technology Assessment undertaken for the National Institute for Health Research [19] concluded that use of NILT was more cost-effective than liver biopsy in detecting cases of advanced fibrosis. Tapper et al. [58] demonstrated that the use of the NAFLD fibrosis score and fibroscan in primary care yielded cost-effective results. Robust data are lacking regarding the performance of PCP’s in the identification of patients with advanced liver disease. Additionally, there is no published randomised controlled trial exploring the performance of NILT in primary care. Whilst limiting the model, the information provided by the analysis may be supplemented with the results from real-life pilot studies in due course. Harman et al. [59] have demonstrated the use of fibroscan in patients with risk factors for CLD, including diabetes, obesity and alcohol excess can increase detection of cirrhosis by 140%, similar to the modelling results in scenario 5 (132%). Our group has evaluated the pathway employing FIB-4 and ELF (Scenario 2) [13].

## Conclusions

This study demonstrates that the introduction of NILT in primary care has the potential to increase the detection of cases of NAFLD with advanced fibrosis and cirrhosis, reduce unnecessary referrals to secondary care of patients at low risk of liver disease and to deliver immediate and sustained significant cost savings. The model provides compelling evidence for clinicians, commissioners and policy makers to consider the formal introduction of non-invasive liver fibrosis testing in primary care, in line with other central policy statements [4, 60, 61].

## Data Availability

The datasets used and analysed during the current study are available from the corresponding author on reasonable request.

## Abbreviations

ALD: Alcoholic liver disease
ALT: Alanine aminotransferase
AST: Aspartate aminotransferase
BCLC: Barcelona Clinic Liver Cancer
CLD: Chronic liver disease
ELF: Enhanced Liver Fibrosis
FIB-4: Fibrosis-4 score
HCC: Hepatocellular carcinoma
HCV: Hepatitis C Virus
LFT: Liver function tests
NAFLD: Non-alcoholic fatty liver disease
NHS: National health system
NILT: Non-invasive liver fibrosis tests
PCP: Primary care physician
SOC: Standard of care
